# Dying with behavioral and psychological symptoms of dementia in Australian nursing homes: a retrospective case-control study

**DOI:** 10.3389/fpsyt.2023.1091771

**Published:** 2023-05-15

**Authors:** Peter Roach, Melanie R. Lovell, Stephen Macfarlane

**Affiliations:** ^1^Greenwich Hospital, HammondCare, Greenwich, NSW, Australia; ^2^Mona Vale Hospital, Northern Sydney Local Health District, Mona Vale, NSW, Australia; ^3^Northern Clinical School, Sydney Medical School, University of Sydney, St Leonards, NSW, Australia; ^4^The Dementia Centre, HammondCare, St Leonards, NSW, Australia; ^5^Faculty of Medicine, Nursing and Health Sciences, Monash University, Clayton, VIC, Australia

**Keywords:** dementia, palliative care, end-of-life, behavior, nursing homes, BPSD, prognosis, mortality predictors

## Abstract

**Objectives:**

To identify predictors of mortality in people with active and challenging behavioral and psychological symptoms of dementia (BPSD).

**Design:**

A retrospective case–control study was designed to compare those referred to Dementia Support Australia (DSA) who died in the 12 months to November 2016, with an equal number of controls who did not die. An audit tool was designed after literature review and expert opinion from the service. Odds ratio calculations and the Mann–Whitney U test were used to assess for difference.

**Setting:**

Residents of Australian residential aged care facilities with BPSD referred to the DSA service.

**Participants:**

Of 476 patients referred to DSA during the study period, 44 died. 44 controls were randomly selected from those remaining matched for age and sex.

**Results:**

Significant differences included higher rates of benzodiazepine use, drowsiness, delirium, reduced oral intake and discussions about goals of care in those who died. Those who died were referred to the service for a shorter period and had more frequent contact between DSA and nurses at the nursing homes. Increase in opioid use and loss of skin integrity in those who died approached significance. The overall end of life course demonstrated a complex set of needs with frequent delirium, pain and frailty.

**Conclusion:**

Further study is required to determine the optimal care for those with BPSD at the end of their lives. This study would indicate complex end of life care needs and point to a role for palliative care support.

## 1. Introduction

Dementia is an incurable, fatal illness whose prevalence is increasing in Australia. In 2016 dementia became the second leading cause of death after ischemic heart disease, and the leading cause of death in women (1). Despite this, people with dementia have rarely been referred for palliative care which has traditionally been focused on people with cancer (2). Core domains of optimal palliative care in dementia were therefore defined comprising of symptom control and comfort (3). However there is limited high level evidence to support palliative care interventions (4, 5), and research is needed.

Behavioral and psychological symptoms of dementia (BPSD) is a term that includes affective symptoms (depression and apathy), agitation, wandering, verbalization, sleep problems, hallucinations and delusions (6). The prevalence rate in those with dementia is high, in some studies almost universal (7, 8). It appears some categories of BPSD tend to increase as cognitive function worsens, particularly psychosis, agitation, hyperactivity and physical aggression, but others such as apathy do not (4).

Predictors of mortality and end of life care needs in the subgroup of patients with particularly active and challenging BPSD, for instance physical agitation and aggression, sexualized behavior and repeated wandering is not well studied (9). The prevalence of these symptoms is variable in different studies, with a systematic review putting physical agitation at an incidence of 19–80% (6). Even the lower end of this range will make the more active symptoms of BPSD an important issue given the rising prevalence of dementia in the population. These active symptoms, where patients tend to transgress social norms, are also particularly distressing for caregivers (10).

This study reviewed patients referred to Dementia Support Australia (DSA) over 12 months to November 2016. DSA is a service for patients with challenging BPSD from across Australia. The interventions carried out by DSA focus on non-pharmacological management (see Table 1) (11). The aims of the study were to review the characteristics of those dying with this form of challenging BPSD. This information would help identify those with BPSD at higher risk of dying, better understand their needs at the end of their lives and identify those who may benefit from referral to specialist palliative care.

**Table 1:** General pattern of DSA intervention.

General pattern of DSA intervention
Admit the patient and gather information
Assess for reversible factors that may be leading to a delirium
Assess the behavior, including types and triggers
Assess for pain
Assess the patient’s psychosocial history including contact with families or carers in all cases and the patient’s GP
Visit the site (average 3.6 site visits per patient in the case group, 4.3 in the control group) on different days, weekends and evenings
Minimize use of restraints
Review medications, in some patients obtaining a specialist psychogeriatric opinion via phone or email
Educate staff on management of patients with behavioral and psychological symptoms of dementia (BPSD)
Form an individual management plan for the patient reflecting their psychosocial history, current symptoms and the nursing home environment
Provide diversional equipment and at times one to one nursing support
Follow up the patient and nursing home staff with site visits and calls or emails
Discharge the patient once the nursing home is managing with the patient behavior, or the patient is transferred to hospital, or the patient dies

**Table 2:** Characteristics of the patient groups.

	Died (*n* = 44)	Discharged (*n* = 44)	Odds ratio (95% CI)
Male:Female	24:20	24:20	
Mean age (years)	80.2	77.1	
Time had lived at the nursing home prior to referral (months)	10.3	10.2	
Co-morbidities (%)			
Cardiovascular disease	77	64	1.94 (0.76–4.95)
Diabetes	27	14	2.38 (0.80–7.04)
Mental health diagnosis (eg: depression/anxiety)	68	59	1.48 (0.62–3.55)
Osteoarthritis	56	41	1.90 (0.82–4.43)
Malignancy	13.6	4.5	3.32 (0.63–17.43)
Dementia type (%)			
Alzheimer’s	57	55	1.10 (0.47–2.54)
Mixed dementia	18	6.8	3.04 (0.75–12.32)
Vascular dementia	14	16	0.83 (0.26–2.72)
Lewy body dementia	4.4	6.8	0.65 (0.10–4.10)
Frontotemporal	6.8	4.4	1.54 (0.24–9.68)
Alcohol	2.2	2.2	N/A
Medications (%) and most common prescribed			
Antipsychotics	84 (risperidone)	80 (risperidone)	1.36 (0.46–4.04)
Benzodiazepines	80 (oxazepam)	41 (oxazepam)	5.62 (2.17–14.48)
Opioids	66 (buprenorphine)	45 (buprenorphine)	2.32 (0.98–5.49)
Antidepressants	57 (mirtazapine)	45 (sertraline)	1.58 (0.68–3.66)
Anticholinesterase inhibitors/NMDA antagonists	11 (donepezil)	14 (donepezil)	0.81 (0.23–2.89)
Statins	18	18	N/A
Aspirin or clopidogrel	41	23	2.35 (0.93–5.94)
Mood stabilisers	11 (valproate)	34 (valproate)	0.25 (0.81–0.76)
Fall documented (%)	64	45	2.10 (0.89–4.93)
Skin wounds/pressure areas (%)	43	23	2.58 (1.03–6.51)
Hospitalization (%)	48	52	0.83 (0.36–1.92)
Days admitted (mean)	6.95	5.45	*p = 0.98*
Antibiotic use (%)	45	31	1.79 (0.75–4.26)
Numbers of medications prescribed (mean)	7.95	6.93	*p = 0.19*

## 2. Study

### 2.1. Methods

A retrospective case–control study was conducted. Of 476 patients referred to DSA during the study period, the 44 patients who died and 44 randomly selected sex-matched controls (24 men and 20 women) were included. The groups were then checked to ensure average age was not a significant difference between the two groups (80 years in the cases, 77 years in the controls, *p* = 0.1).

Data was extracted using an audit tool developed for the study. Data items were informed by literature review and expert recommendation from the service. Data was de-identified. Data sources included the original referral (nursing progress notes, medication charts, behavior charts, general practitioner referral letters), funding received through the service (for example for diversional therapy tools and one to one nursing), records of each site visit, each telephone call and email, and letters produced by the service with their management suggestions.

The presence of delirium, drowsiness, use of antibiotics, skin integrity, hospitalization and other characteristics of the two groups were ascertained by reading the assessments of staff working for DSA in their documentation. The overall progress of the patients during their time with the service was also examined in this way. Behavior tools were a part of each record and contained a count of challenging behaviors during the period divided into subtypes: physical, verbal and wandering.

Binary outcomes were analyzed using a standard odds ratio and confidence interval calculation. Non-binary outcomes were compared between the two groups using the Mann–Whitney U rank test (12).

Ethics approval was sought and given by the Northern Sydney Local Health District Human Research Ethics Committee as a low/negligible risk project (HREC reference: LNR/17/HAWKE/328).

### 2.2. Results

Results are summarized in Tables 1–3. There were a number of similarities between the cases and controls and this demonstrates the challenge of trying to identify those dying. Both groups had been residents at their nursing homes for similar periods of time (cases 10.3 months, controls 10.8 months). They had common background rates of cardiovascular disease (77 and 64%), mental health diagnoses (68 and 59%) and subtypes of dementia (Alzheimer’s at 57 and 55%). The other subtypes of dementia in descending order in both groups were mixed dementia, vascular dementia and Lewy Body disease.

**Table 3:** Significant predictors of mortality.

Characteristics associated with those with BPSD who died				
	Died (*n* = 44)	Discharged (*n* = 44)	Odds ratio (95% confidence interval)	*p* value
Regular benzodiazepine	35	18	OR = 5.617 (2.17–14.48)	*p* = 0.0004
Drowsiness	35	21	OR = 4.23 (1.65–10.8)	*p* = 0.003
Delirium diagnosed	21	9	OR = 3.55 (1.39–9.10)	*p* = 0.008
Discussions about goals of care	27	4	OR = 15.9 (4.85–52.45)	*p* < 0.0001
Reduced oral intake	32	12	OR = 10.4 (3.88–27.93)	*p* < 0.0001

Health service interventions associated with those with BPSD who died			
	Died	Discharged	*p* value
Contact to and from DSA per day	1.15	0.82	*p* = 0.0027
Average calls from nursing staff at nursing homes to DSA per day	0.12	0.03	*p* < 0.001
Average calls from DSA to nursing staff per day	0.38	0.27	*p* = 0.01
Total numbers of documented behaviors per month	70.7	55.6	*p* = 0.036
Time admitted to service (days)	49.9	72.4	*p* = 0.02
Dollars spent on supplemental nursing and diversional tools ($)	2064	695	*p* = 0.02

Both groups were frequently prescribed anti-psychotic (cases 84%, controls 80%), opioid and benzodiazepine medications. Risperidone, buprenorphine patches and oxazepam were the most frequently prescribed in each class. Falls were common, as were skin wounds or pressure areas. Hospitalization was similar between the groups (48% of cases and 52% of controls). The most frequent cause of hospitalization in both groups was behavioral disturbance. Antibiotic use occurred in 45% of the group who died and 31% of the controls, most commonly for urinary tract and chest infections. Both groups had similar rates of incontinence (61% in both groups). Pain was common (86% of cases and 73% of controls), with low rates of documented use of pain scales (16% cases and 18% controls). Pain was most commonly attributed to osteoarthritis.

Nursing home staff injuries were common (82% cases, 75% controls) and at times severe. One staff member developed a “collapsed lung” and was off work due to being punched. A fellow resident developed a fractured arm from being pushed. Sexualized behavior toward staff was frequently documented. The patients themselves appeared distressed in the descriptions of the behaviors. One man had taken to crawling around the unit on his hands and knees calling out for help. Patients appeared to fear personal care (resistance to care was 98% in those who died and 93% in controls) and the use of hoists. Being afraid, wanting to go home and wanting to find family members were all frequently documented.

82% of those who died did so in the nursing home, the remainder dying in hospital. 4% of the cases and 2% of the controls were referred to palliative care. Dying was challenging to recognize and was either not recognized at all (36%), or only recognized in the final days of life (39%). The most frequent causes of death stated were dementia and infection.

There were significant differences between cases and controls Table 3. Delirium was diagnosed in a significantly higher number of those who died (48%) compared with the controls (20%) (*p* = 0.008). There were limited formal scores and it seemed to be a clinical diagnosis made, often on whether reversible factors were present (eg: infections) and whether the patient’s condition fluctuated. The sleep–wake cycle was frequently disturbed in patients across both groups (98% of those who died, 86% of those discharged). Documented daytime drowsiness was significantly higher in those who died at 80% compared with the controls at 48% (*p* = 0.003).

Documented reduced oral intake was significantly higher in those dying compared with the controls (*p* < 0.001).

Behavioral disturbance was assessed by behavior charts or nursing progress notes. 70% of the patients who died and 84% of those discharged had sufficient documentation to allow assessment and behaviors were divided into physical (eg: biting, hitting), verbal (swearing, shouting) and wandering. Patients who died had higher numbers of recorded behavioral disturbances that those who were discharged (70.7 compared with 55.6, *p* = 0.036).

Total calls, emails and documentation to and from DSA, averaged out for the duration of referral to the service, were significantly higher in the group who died compared to the group discharged (*p* < 0.001). This was the result of significantly higher contact both from DSA to the nursing home staff (*p* = 0.01), and from the nurses at the nursing homes back to DSA (*p* < 0.001), indicating greater staff concern in this group. 11% of those who died were referred to specialist palliative care.

The main medication that was significantly more prescribed in those who died were benzodiazepines (midazolam was excluded to avoid this being confounded by use in those more clearly dying). 35 of the cases and 18 of the controls were prescribed regular benzodiazepines (OR 5.62 [CI 2.17–14.48]). The most frequent agent was oxazepam.

A discussion about goals of care or documentation of an advance care plan was significantly more likely to have occurred in the group who died compared with the group who were discharged (61% compared with 9%, OR = 15.9 [CI = 4.85–52.45]) which would seem to indicate a concern about prognosis.

The striking difference exhibited by those who died was a change in behavior in the final weeks of life. A common pattern was intermittent daytime drowsiness and reduced oral intake in the short weeks leading up to death. In between episodes of drowsiness patients did at times continue to have episodes of severe BPSD though generally there was a shift to the severity reducing. Although oral intake almost always reduced leading up to death, one of the patterns was missed meals due to drowsiness, with good intake at other meals. Others did have a more consistently reduced oral intake. It appeared dying was challenging to predict in some patients due to these intermittent periods of good oral intake and being alert, interspersed with period of ongoing behavioral disturbance.

### 2.3. Discussion

In this case–control study, the key differences in those dying with BPSD and those who did not die were the presence of delirium and drowsiness, reduced oral intake, benzodiazepine use and nursing concern.

Studies have looked at characteristics associated with dying in those with dementia (13, 14), though studies analyzing those with BPSD are more limited. Benzodiazepines have been linked with increased mortality in those with dementia (13). They are not recommended routinely for BPSD however their use is widespread both in the general community and in nursing homes (15). Given the evidence that benzodiazepines may have limited overall benefit in treating BPSD symptoms (16), this study would support the need to carefully review benzodiazepine use in patients with BPSD, seeking to minimize their use where possible.

Opioids were more frequently prescribed to the group who died, although the difference was not statistically significant, and pain was common in both groups. The diagnosis and management of pain in people with BPSD are important discussion points. The Abbey pain scale is a validated tool to help determine whether those with dementia who cannot express their pain are experiencing it (17). The Abbey scale was frequently recommended but infrequently used by the nursing homes caring for patients. Pain was frequently reported in the patients in this study, similar to other studies of dementia patients nearing the end of life, with evidence their incidence of pain rises to levels similar to those seen in patients with cancer (18, 19). This study would suggest a role for the greater use of a validated scale such as the Abbey pain scale in those with dementia, both in the diagnosis and monitoring of pain.

Delirium and drowsiness were both more frequently present in those who died. Although there were no formal delirium scores, the fluctuating nature of the drowsiness, oral intake and behavior in those dying makes it likely that delirium was under-diagnosed in that group. Diagnosing delirium superimposed on dementia is challenging, however fluctuating motor performance and levels of alertness or arousal are considered key markers of delirium in those with dementia (20). It would seem from this study that many in the case group had delirium in the last weeks leading up to death. Studies have shown higher mortality in the aged with delirium (21) and in those with dementia with delirium (22). The presence of delirium in palliative care studies has been linked with higher rates of symptom expression (23). This study would support the presence of delirium, particularly where there is no reversible cause, as being a reason to prepare families for the possibility of dying and to discuss goals of care.

Another finding from this study was the significantly higher contact between DSA and nurses at the nursing homes in the group who died. Another study has shown a link between an unscheduled GP visit and dying (24). This study, though, would suggest a correlation between dying and concern about the patient from both nursing staff and the service. Even though dying was not recognized in all patients, the trajectory of their condition tended to prompt increased follow up and contact. This pattern could help identify those at risk of dying, by flagging those patients about whom nurses are particularly concerned.

Lastly, symptoms of frailty were common in both groups and not significantly different between them. There was insufficient data to calculate body mass index which has been inversely correlated with increased mortality in patients with dementia in nursing homes (25). Presence of pressure injuries neared significance, and increased mortality in those with pressure injuries would be consistent with prior work (26). Overall frailty was a common feature of both groups, and loss of skin integrity should be a potential alert to an increased risk of dying.

Only a small number of patients who died (11%) were referred to specialist palliative care services. A significant issue is a lack of clear criteria to trigger a palliative care referral in those with dementia (27) and this needs significant work given the prevalence of dementia in many countries with aging populations. Given the symptoms and needs in the patients reviewed in this study, specialist palliative care would appear to have a role in managing symptoms and helping with advance care planning.

This study had several limitations. It was retrospective in its design and not blinded, opening the study to the possibility of bias. Several of the areas associated with death in this study, for instance the behavior count, were obtained from non-standardized charts with variable detail. Prospective studies to gain greater knowledge of those with BPSD would be worthwhile, with standardized methods of measuring pain and behavioral disturbance. This could then lead to interventions in blinded, randomized controlled trials, developing a larger evidence base to guide the management of those with BPSD. The study involved small numbers of patients and should therefore be interpreted with caution, with larger studies being needed. The study was also restricted to Australian nursing homes.

This study has identified several areas that could alert clinicians to the possibility of someone with BPSD being at risk of dying. Additionally, those dying with BPSD would appear to have significant palliative care needs, most obviously symptom management (physical and psychological) and advance care planning. The detailed psychological and behavioral approach combined with nursing staff education, typified by services such as DSA, have evidence supporting their approach (28, 29). An assessment of the palliative care needs of patients with the risk factors identified in this study should be considered to help manage the needs of those dying, along with specialist palliative care referral where complex symptoms or more challenging advance care planning discussions are required.

## Data availability statement

The datasets presented in this article are not readily available because as part of the ethics application the data access is restricted. Requests to access the datasets should be directed to proach@hammond.com.au.

## Ethics statement

The studies involving human participants were reviewed and approved by Northern Sydney Local Health District. Written informed consent for participation was not required for this study in accordance with the national legislation and the institutional requirements.

## Author contributions

PR performed data collection, statistical analysis, and the first draft of the manuscript. ML and SM analyzed the data and results. PR, ML, and SM were involved in study concept and design and critical revision of the manuscript. All authors contributed to the article and approved the submitted version.

## Conflict of interest

PR, ML, and SM were employed by HammondCare.

The authors declare that the research was conducted in the absence of any commercial or financial relationships that could be construed as a potential conflict of interest.

The reviewer CP declared a past collaboration with one of the authors SM to the handling editor.

## Publisher’s note

All claims expressed in this article are solely those of the authors and do not necessarily represent those of their affiliated organizations, or those of the publisher, the editors and the reviewers. Any product that may be evaluated in this article, or claim that may be made by its manufacturer, is not guaranteed or endorsed by the publisher.
